# Proteome changes of fibroblasts and endothelial cells upon incubation with human cytomegalovirus subviral Dense Bodies

**DOI:** 10.1038/s41597-023-02418-2

**Published:** 2023-08-04

**Authors:** Inessa Penner, Mario Dejung, Anja Freiwald, Falk Butter, Jia-Xuan Chen, Bodo Plachter

**Affiliations:** 1 Institute for Virology, University Medical Center of the Johannes Gutenberg-University, Mainz, Germany; 2 Institute of Molecular Biology, Johannes Gutenberg-University, Mainz, Germany

**Keywords:** Herpes virus, Viral infection

## Abstract

Human cytomegalovirus (HCMV) is a pathogen of high medical relevance. Subviral Dense Bodies (DB) were developed as a vaccine candidate to ameliorate the severe consequences of HCMV infection. Development of such a candidate vaccine for human application requires detailed knowledge of its interaction with the host. A comprehensive mass spectrometry (MS)- based analysis was performed regarding the changes in the proteome of cell culture cells, exposed to DB.

## Background & Summary

Human cytomegalovirus (HCMV) is a β-herpesvirus and the leading cause of congenital infections worldwide, resulting in a number of sequelae such as hearing loss, visual deficits or cognitive disorders^1^. In the context of systemic immunosuppression, HCMV infection can result in substantial morbidity and mortality^1^. Fibroblasts, infected with HCMV release high amounts of non-infectious particles, termed Dense Bodies into the cell culture supernatant^2,3^. Mass spectrometry analyses of isolated DB have contributed to the elucidation of their protein composition and revealed the presence of important antigens of the adaptive immune response against HCMV^4,5^. Meanwhile, *in vitro* experiments and animal studies showed that DB are notably immunogenic and induce a robust interferon (IFN) response^6–11^. Consequently, DB have been considered as a promising vaccine against HCMV^12,13^. With regard to human application of a candidate vaccine in clinical trials and for ultimate licensing, comprehensive knowledge about the impact of the vaccine on host cells is important to assess potential adverse effects and tolerability. Data sets on the impact of DB on culture fibroblasts and endothelial cells were generated using mass spectrometry (MS). These datasets were used in a previous publication that focused on the investigation of the Interferon-β response and the induction of Interferon-stimulated gene (ISG) expression in fibroblasts and endothelial cells upon DB exposure^11^.

## Methods

### Cell culture

Primary human foreskin fibroblasts (HFF) were established from the foreskin of a newborn child in 1994 and were used for research in the past^6,7,14–16^. Approval to use these cells for the studies was obtained from the ethics committee of the medical council of Rheineland-Palatinate, Germany. HFF were maintained in minimal essential medium (MEM; Gibco-BRL, Glasgow, Scotland) supplemented with 5% fetal calf serum (FCS), 100 mg/l L-glutamine, 0.5 ng/ml basic fibroblast growth factor (bFGF, Invitrogen, Karlsruhe, Germany) and gentamicin (5 mg/l). For the experiments, HFF cell passage numbers between 16 to 19 were used. HEC-LTT cells were established by Dagmar Wirth and coworkers^17^. The cells were derived from human umbilical vein endothelial cells (HUVECs) that were conditionally immortalized with tetracycline-dependent expression of the SV40 large-T antigen and human telomerase reverse transcriptase (hTERT). For cultivation, culture vessels were coated with 0.1% gelatin (Sigma-Aldrich, Saint Louis, MO;) for at least 30 minutes. HEC-LTT cells were maintained in endothelial growth medium (EGM BulletKit; Lonza Sales Ltd., Basel, Switzerland) supplemented with 2 μg/mL doxycycline (Sigma-Aldrich, Saint Louis, MO). The proliferation of HEC-LTT cells can be controlled by doxycycline (DOX). The addition of DOX activates the expression of the immortalizing proteins hTERT and SV40 large-T antigen, resulting in cell proliferation. Doxycycline was omitted during the entire experiments. Permission to use HEC-LTT cells for research purposes was granted via a material transfer agreement by the Helmholtz Centre for Infection Research (HZI). The cells were shown to be permissive to HCMV infection^18^. HEC-LTT were kindly sent to us by Christian Sinzger (Institute for Virology, Ulm University Medical Center, Ulm, Germany) and used for experiments from passage 41 to passage 55.

### Preparation of virus seed stocks

Virus seed stocks were prepared from supernatants of transfected HFF. Briefly, the strain Towne-repΔGFP (hereafter denoted as TR-∆GFP) was reconstituted by transfection of bacterial artificial chromosome (BAC) DNA, containing the Towne-repΔGFP genome into HFF. The generation of the BAC clone was described Lehmann *et al*.^7^. Transfected cells were propagated until 100% of the cells showed cytopathic effects (CPE). The virus-containing supernatants from these cultures were harvested and precleared from cellular debris by centrifugation at 1,475 × g for 10 min and then stored as virus seed stocks at −80 °C for further propagation of the virus.

### Generation of experimental stocks

Virus experimental stocks were prepared from supernatants of HFF, infected with virus seed stocks. For this, HFF were seeded at a density of 1.8 × 10^6^ in five 175 cm^2^ tissue culture flasks and infected with 5 ml virus inoculum per flask. For this, 1 ml of the TR-∆GFP seed stock-supernatant and 4 ml of 5% MEM medium were mixed and added to the cells for 1.5 hours. Then 15 ml of fresh 5% MEM medium was added and the infected cells were incubated at 37 °C until the cultures showed a complete CPE. The cell culture supernatants were harvested and combined. Cellular debris was removed by centrifugation at 1,475 × g for 10 min at room temperature. Finally, the supernatants were stored in freezing tubes at −80 °C.

### Preparation of HCMV dense bodies

For the purification of Dense Bodies, twenty 175 cm^2^ tissue culture flasks with 1,8 × 10^6^ HFF were infected with 1 ml of frozen virus supernatant stocks of the HCMV strain TR-ΔGFP, diluted in 4 ml 5% MEM medium. Following virus adsorption for 1.5 h, 15 ml of fresh 5% MEM medium, supplemented with 50 nM of Letermovir (LMV) were added and HFF were incubated for at least 7 days. LMV was added to the cell culture media every 3 days after initial infection. LMV is a highly specific inhibitor of the HCMV terminase complex and was shown to inhibit HCMV replication in cell culture by interfering with the cleavage/packaging of HCMV genomes into nuclear capsids^19^. Supernatants from infected HFF that showed a complete cytopathogenic effect (CPE) were harvested and gross cellular debris was removed by centrifugation for 10 min at 1,475 × g. Afterwards viral particles were pelleted via ultracentrifugation at 95.000 × g for 70 min at 10 °C using a 45Ti rotor in a Beckman Optima L-90K ultracentrifuge. For fractionation of the particles, the pellets were resuspended in 2 ml of phosphate-buffered saline (PBS) and loaded onto glycerol-tartrate density gradients. For gradient preparation, 5 ml of a 35% Na-tartrate solution in 0.04 M Na-phosphate buffer, pH 7.4 and 4 ml of a 15% Na-tartrate–30% glycerol solution in 0.04 M Na-phosphate buffer, pH 7.4 were mixed in a gradient mixer and introduced into a polycarbonate centrifuge tube (14 ml; Beckman Ultra-Clear centrifuge tubes) at an angle of 45°. Gradients were overlaid with 1 ml of the concentrated viral particle suspension and centrifuged in a Beckman SW41Ti swing-out rotor for 60 min at 90,000 × *g* and 10 °C without deceleration. Subsequently, the DB-fraction was visualized by light scattering and collected by puncturing the tube with a syringe. The fraction collected from gradients containing DB were washed with 10 ml PBS and DB were concentrated by ultracentrifugation using a SW41Ti swing-out rotor for 90 min at 98,000 × *g* and 10 °C. Finally, the DB-pellet was resuspended in 250 µl PBS. Aliquots of 30 µl were prepared and stored at −80 °C until further use. For the determination of DB-protein concentrations, the Pierce™ BCA Protein Assay Kit (23225, ThermoFisher Scientific, Darmstadt, Germany) was used according the manufacturers protocol.

DB were thawed and irradiated with ultra-violet (UV) light shortly before they were applied to cells. Following resuspension in a total volume of 120 µl PBS, DB were transferred onto a spot plate and UV-irradiated at a wavelength of 254 nm for 2 minutes. Then, 100 µl of the UV-irradiated DB/PBS solution were mixed with 2,9 ml culture medium and added to the cells.

### Preparation of protein extracts for proteomic analysis

HFF were seeded at a density of 0.5 × 10^6^ in two 10 cm dishes. On the next day, 20 µg of TR-∆GFP- derived DB were UV-irradiated and added to each dish. The DB-inoculum was incubated for 2 h. Afterwards, 7 ml MEM medium was added and cells were incubated for additional 22 h. Next, the medium was removed and the cells were washed twice with PBS. The HFF of two dishes were pooled and the cell number was determined. 1 × 10^6^ fibroblasts were lysed in 40 µl 2x Laemmli buffer without bromophenol-blue staining and heated at 99 °C for 10 min. After cooling, NuPAGE LDS Sample Buffer (4x) (Life technologies) and 100 mM DTT were added and the samples were incubated at 70 °C for further 10 min.

Endothelial cells were seeded at a density of 0.6 × 10^6^ in two 10 cm dishes in absence of doxycycline. ECs were exposed to 40 µg of UV-irradiated DB of the HCMV strain TR-∆GFP. The following steps were performed as described for HFF.

### Protein in-gel digestion

Proteins were loaded onto a 10% NuPAGE Bis-Tris gel and resolved briefly. Following that, the gel was stained with Coomassie blue and cut into small cubes using a clean scalpel. Gel destaining was performed in 50% ethanol/25 mM ammonium bicarbonate. Protein reduction was done in 10 mM DTT at 56 °C, followed by alkylation in 50 mM iodoacetamide in the dark at room temperature. Trypsin (1 µg per sample) was used to digest the proteins in 50 mM TEAB (triethylammonium bicarbonate) buffer overnight at 37 °C. Peptide extraction was performed sequentially in 30% and 100% acetonitrile. Thereafter, the sample volume was reduced in a centrifugal evaporator to remove residual acetonitrile. Then, the sample was filled with 100 mM TEAB to reach a final sample volume of 100 µl.

### Dimethyl-labelling

According to the experimental design scheme^20^, the digested samples were labelled as “Light”, “Medium” or “Heavy” by adding 4 µl of 4% formaldehyde, formaldehyde-d_2_ or formaldehyde-^13^C, d_2_ solution, respectively. This was then followed by addition of 4 µl of 0.6 M NaBH_3_CN (to “Light” or “Medium” sample) or NaBD_3_CN (to “Heavy” sample). Thereafter, the samples were incubated at room temperature with orbital shaking for 1 h. The labelling reaction was then quenched by adding 19 µl of 1 M ammonium bicarbonate (final concentration 150 mM) and incubated at room temperature with orbital shaking for 15 min. Afterwards, peptides were acidified with formic acid to reach pH ~3. The paired labelled samples were then combined. The resultant peptide solution was purified by solid phase extraction in C18 StageTips^21^).

### Lqiuid chromatography tandem mass spectrometry

Peptides were separated in an in-house packed 30-cm analytical column (inner diameter: 75 μm; ReproSil-Pur 120 C18-AQ 1.9-μm beads, Dr. Maisch GmbH; heated at 40 °C) by online reversed phase chromatography through a 225-min non-linear gradient of 1.6–32% acetonitrile with 0.1% formic acid at a nanoflow rate of 225 nl/min. The eluted peptides were sprayed directly by electrospray ionization into a Q Exactive Plus Orbitrap mass spectrometer (Thermo Scientific). Data-dependent acquisition was carried out using a top10 method. Following each full scan (mass range: 300 to 1,650 *m/z*; resolution: 70,000, target value: 3 × 10^6^, maximum injection time: 20 ms), up to 10 MS2 scans were performed via higher energy collision dissociation (normalised collision energy: 25%, resolution: 17,500, target value: 1 × 10^5^, maximum injection time: 120 ms, isolation window: 1.8 m/z). Charge state selection was performed by rejecting precursor ions of unassigned or +1 charge state. Dynamic exclusion time was set to 35 s.

### Mass spectrometry data processing and statistical analysis

Raw data files were processed by MaxQuant software package (version 2.1.3.0)^22^ using Andromeda search engine^23^. Spectral data were searched against a target-decoy database consisting of the forward and reverse sequences of UniProt proteomes downloaded on 10^th^ August 2022 listed for the specific Taxon IDs (ID 9606, H. sapiens, 79759 entries; ID 10359, HCMV, 17993 entries; ID 10363, HCMV Town strain, 304 entries) and a list of 246 common contaminants. Corresponding dimethyl labels were assigned as “Light” (DimethLys0 and DimethNter0), “Medium” (DimethLys4 and DimethNter4) and “Heavy” (DimethLys8 and DimethNter8) according to the labelling scheme. For each peptide, up to 3 labelled amino acids were allowed. Trypsin/P was chosen for enzyme specificity. Carbamidomethylation of cysteine was selected as fixed modification. Protein N-terminus acetylation and oxidation of methionine were assigned in variable modifications. Up to 2 missed cleavages were tolerated. A minimum peptide length of 7 amino acids was required. For both peptide and protein identifications, a false discovery rate (FDR) of 1% was chosen.

For protein quantification, minimum ratio count was set to one. Both the unique and razor peptides were used for quantification. The “re-quantify” function was switched on. The “advanced ratio estimation” option was also chosen. Reverse hits and potential contaminants were filtered out. Protein groups with at least one unique peptide were retained. Ratios of label-swapped samples were inverted to represent dense bodies treatment (DB) over control for all technical and biological replicates. The normalized ratios were then log2 transformed and median-centered. Afterwards, the ratios of the technical replicates belonging to the same biological replicate were averaged ignoring the missing value if present.

Following the above-mentioned steps, statistical analysis to identify differentially-regulated proteins was performed using the limma software package in R^24^. For fibroblasts, proteins with ratios in at least three out of five biological replicates were retained. For endothelial cells, proteins with ratios in at least two biological replicates were retained. A linear model was then fitted to assess the ratios for each protein without further adjustment for multiple testing. The log2 fold change and the significance of the difference were displayed in a volcano plot. Only proteins with a minimum log2 fold change of 1 and a p value lower than 0.05 were considered as being differentially regulated.

### Proteome analysis of fibroblasts exposed to dense bodies

In our proteomics study, we investigated the impact of HCMV Dense Bodies (DB) incubation on two different cell types. The changes in the cellular proteome of fibroblast or endothelial cells upon DB application was compared to mock-treated reference samples.

**Fig. 1:**
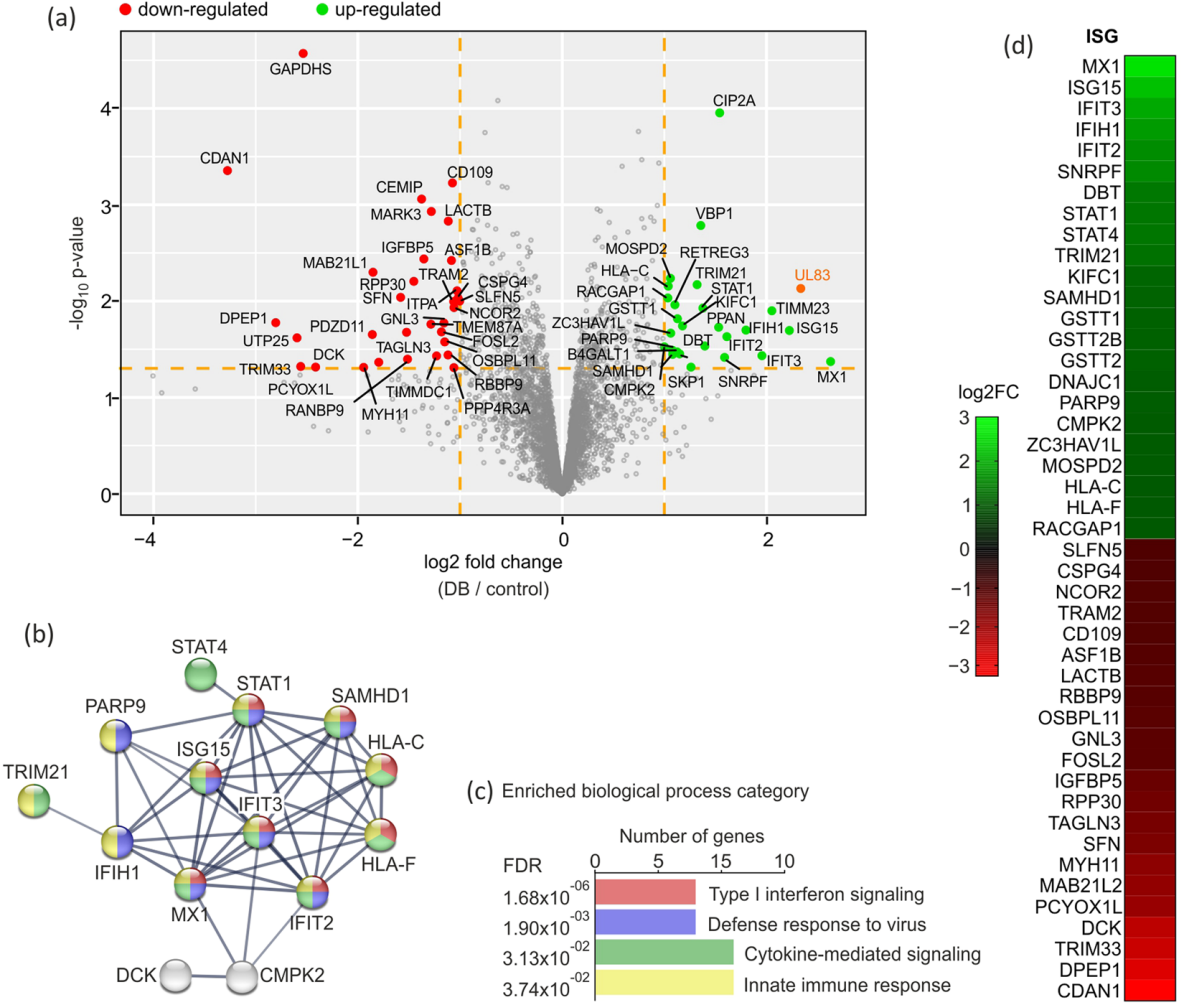
Proteome analysis of DB-regulated proteins in HFF. HFF were mock-treated or incubated with 20 µg of UV-inactivated DB derived from TR-∆GFP. Cell lysates were subjected to total proteome MS/MS analysis at 24 h post DB- application. (**a**) Volcano plot showing the log_2_ fold-change (x-axis) versus the significance (y-axis) of the in total 3757 proteins, detected in five biological replicates. The dotted lines in orange show the cut-off fold change of ±1.0 and a p-value of 0.05. The significance (non-adjusted p-value) and the fold-change are converted to −log10(p-value) and log2 fold-change, respectively. There were 33 proteins increased by >1.0-fold with p-value < 0.05 (green dots), and 35 proteins that were decreased by < − 1.0-fold with p-value < 0.05, (red dots). The HCMV tegument protein pp65 (UL83) is highlighted in orange and its detection was used as a positive control, indicating DB internalisation into HFF. The volcano plot was generated using the R software. (b + c) Protein-Protein Interaction (PPI) analysis and functional classification of the regulated proteins in HFF after DB-stimulation. (**b**) Display of the STRING PPI network generated upon entering the 68 regulated proteins into the STRING database. The network nodes represent all the proteins produced by a single protein-coding gene locus. Nodes are coloured according to their function in the indicated biological processes in c. Grey nodes indicate proteins connected to the input proteins but without association with the biological processes. Connections reflect protein interaction and the line thickness indicates strength of the data support, using a high confidence cut-off with a score of 0.7. Proteins with no interaction to other proteins in the network were removed. (**c**) Bar chart of the biological processes, connected to the proteins that were found to be regulated in HFF after DB-stimulation. The arrangement was performed according to increasing False Discovery Rates (FDR). The y-axis represents biological process categories, while the x-axis indicates the number of genes involved in each category. (**d**) Heatmap of the 45 altered ISGs. The expression patterns were arranged hierarchically based on the mean of the log2 converted normalized ratio from 5 biological replicates. The log_2_FC is represented with a colour gradient. IFN, Interferon; ISG, Interferon-stimulated gene; STRING, Search Tool for the Retrieval of Interacting Genes.

Involving five biological replicates, 3757 protein groups were identified in total in fibroblasts after multiple steps of stringency filtering and limma analysis. Using a twofold cut-off, 153 proteins remained, of which 68 showed a p value lower than 0.05. These 68 proteins were considered as being differentially expressed at 24 h post DB-application to fibroblasts and are listed in Table 1. Results are displayed in a volcano plot in Fig. 1a. 33 proteins were upregulated while 35 were downregulated in DB-treated cells in comparison to mock-treated cells. To characterize the relationships between the 68 differentially expressed proteins, the online tool STRING (http://string-db.org/, accessed on 16.05.2023) was applied to analyse the interacting partners. The protein-protein interaction (PPI) network analysis, depicted in Fig. 1b shows one main cluster composed of proteins that function in biological processes of type I interferon response, defense response to virus, response to virus and cytokine-mediated signalling pathways (Fig. 1b). The bar chart in Fig. 1c shows the enriched biological processes arranged according to increasing False Discovery Rates (FDR). Strikingly, when the 68 altered proteins were submitted to the Interferome database online tool (v2.01, accessed on November 2022^25^), the majority (66%) of them were identified as being interferon-stimulated genes (ISGs) (Fig. 1d).

**Table 1:** Differentially expressed proteins identified in response to DB-treatment in fibroblasts.

UniProt ID	Ratio string	Replicate count	Gene name	Protein name	log2ratio					logFC	adjusted p-Value	minus_log10_p_value	significant	ISG*
					repl 1	repl 2	repl 3	repl 4	repl 5					
P20591	42;42;47;36;36	5	**MX1**	Interferon-induced GTP-binding protein Mx1	3.751	−0.911	5.172	4.641	0.486	**2.628**	0.042	1.373	+	yes
Q6SW59	22;22;26;10;10	5	**UL83**	HCMV 65 kDa phosphoprotein	1.544	0.400	4.373	3.210	2.151	**2.336**	0.007	2.132	+	
P05161	12;12;12;7;7	5	**ISG15**	Ubiquitin-like protein ISG15	3.322	−0.084	3.639	3.731	0.512	**2.224**	0.020	1.695	+	yes
O14925	0;0;1;1;1	3	**TIMM23**	Mitochondrial import inner membrane translocase subunit Tim23			0.847	2.641	2.670	**2.053**	0.013	1.901	+	
Q5SRD1	0;0;1;1;1	3	**TIMM23B**	Mitochondrial import inner membrane translocase subunit Tim23B			0.847	2.641	2.670	**2.053**	0.013	1.901	+	
O14879	11;11;14;7;7	5	**IFIT3**	Interferon-induced protein with tetratricopeptide repeats 3	2.111	−0.507	4.355	3.105	0.713	**1.956**	0.037	1.434	+	yes
Q9BYX4	4;4;7;1;1	5	**IFIH1**	Interferon-induced helicase C domain-containing protein 1	1.877	−0.088	2.914	3.536	0.756	**1.799**	0.020	1.699	+	yes
P09913	9;9;19;4;4	5	**IFIT2**	Interferon-induced protein with tetratricopeptide repeats 2	1.778	0.059	3.325	2.528	0.370	**1.612**	0.023	1.631	+	yes
P62306	1;1;3;0;0	3	**SNRPF**	Small nuclear ribonucleoprotein F	2.289	2.240	0.240			**1.590**	0.038	1.416	+	yes
Q8TCG1	1;1;2;0;0	3	**CIP2A**	Protein CIP2A	1.545	1.492	1.589			**1.542**	0.000	3.939	+	
Q9NQ55	2;2;2;0;0	3	**PPAN**	Suppressor of SWI4 1 homolog	0.611	1.632	2.353			**1.532**	0.019	1.729	+	
P11182	1;1;3;0;0	3	**DBT**	Lipoamide acyltransferase component of branched-chain alpha-keto acid dehydrogenase complex, mitochondrial	1.502	2.267	0.420			**1.396**	0.029	1.534	+	yes
P42224	58;58;61;38;38	5	**STAT1**	Signal transducer and activator of transcription 1-alpha/beta	1.979	0.292	2.310	2.010	0.294	**1.377**	0.012	1.928	+	yes
Q14765	58;58;61;38;38	5	**STAT4**	Signal transducer and activator of transcription 4	1.979	0.292	2.310	2.010	0.294	**1.377**	0.012	1.928	+	yes
P61758	4;4;4;1;1	5	**VBP1**	Prefoldin subunit 3	1.294	1.254	0.394	1.985	1.857	**1.357**	0.002	2.785	+	
P19474	3;3;5;2;2	5	**TRIM21**	E3 ubiquitin-protein ligase TRIM21	2.008	0.402	1.974	1.807	0.413	**1.321**	0.007	2.171	+	yes
P63208	1;1;3;1;1	5	**SKP1**	S-phase kinase-associated protein 1	1.909	3.018	1.341	−0.502	0.540	**1.261**	0.048	1.315	+	
Q9BW19	1;1;1;0;0	3	**KIFC1**	Kinesin-like protein KIFC1	1.740	1.331	0.461			**1.178**	0.018	1.742	+	yes
Q9Y3Z3	11;11;24;6;6	5	**SAMHD1**	Deoxynucleoside triphosphate triphosphohydrolase SAMHD1	1.589	−0.339	2.162	2.028	0.310	**1.150**	0.035	1.453	+	yes
P30711	5;5;4;0;0	3	**GSTT1**	Glutathione S-transferase theta-1	1.748	1.056	0.581			**1.129**	0.015	1.816	+	yes
P0CG30	5;5;4;0;0	3	**GSTT2B**	Glutathione S-transferase theta-2B	1.748	1.056	0.581			**1.129**	0.015	1.816	+	yes
P0CG29	5;5;4;0;0	3	**GSTT2**	Glutathione S-transferase theta-2	1.748	1.056	0.581			**1.129**	0.015	1.816	+	yes
P15291	1;1;2;0;0	3	**B4GALT1**	Beta-1,4-galactosyltransferase 1	1.463	1.676	0.237			**1.125**	0.034	1.473	+	
Q86VR2	1;1;1;0;0	3	**RETREG3**	Reticulophagy regulator 3	1.688	0.855	0.767			**1.103**	0.011	1.961	+	
Q96KC8	1;1;1;0;0	3	**DNAJC1**	DnaJ homolog subfamily C member 1	0.358	1.013	1.875			**1.082**	0.036	1.447	+	yes
Q8IXQ6	6;6;8;2;2	5	**PARP9**	Protein mono-ADP-ribosyltransferase PARP9	1.140	−0.416	1.587	2.312	0.774	**1.080**	0.032	1.489	+	yes
Q5EBM0	2;2;5;1;1	5	**CMPK2**	UMP-CMP kinase 2, mitochondrial	0.690	−0.196	1.721	2.084	1.030	**1.066**	0.022	1.666	+	yes
Q96H79	4;4;2;0;0	3	**ZC3HAV1L**	Zinc finger CCCH-type antiviral protein 1-like	1.411	1.431	0.346			**1.063**	0.022	1.667	+	yes
Q8NHP6	2;2;1;0;0	3	**MOSPD2**	Motile sperm domain-containing protein 2	1.497	0.775	0.908			**1.060**	0.006	2.235	+	yes
P10321	5;5;5;4;4	5	**HLA-C**	HLA class I histocompatibility antigen, C alpha chain	1.277	0.122	1.550	1.650	0.593	**1.039**	0.007	2.155	+	yes
P30511	5;5;5;4;4	5	**HLA-F**	HLA class I histocompatibility antigen, alpha chain F	1.277	0.122	1.550	1.650	0.593	**1.039**	0.007	2.155	+	yes
Q9H0H5	3;3;3;0;0	3	**RACGAP1**	Rac GTPase-activating protein 1	1.517	0.945	0.642			**1.035**	0.009	2.031	+	yes
Q16740	2;2;2;0;0	3	**CLPP**	ATP-dependent Clp protease proteolytic subunit, mitochondrial	0.391	1.691	0.919			**1.000**	0.030	1.525	+	
Q08AF3	4;4;5;1;1	5	**SLFN5**	Schlafen family member 5	−0.873	−0.661	−0.705	−2.223	−0.555	**−1.003**	0.010	2.000	+	yes
Q6UVK1	4;4;10;4;4	5	**CSPG4**	Chondroitin sulfate proteoglycan 4	−0.985	−0.626	−0.632	−2.224	−0.631	**−1.019**	0.009	2.031	+	yes
Q9BY32	1;1;1;0;0	3	**ITPA**	Inosine triphosphate pyrophosphatase	−1.110	−1.397	−0.581			**−1.029**	0.008	2.105	+	
Q9Y618	2;2;1;1;1	5	**NCOR2**	Nuclear receptor corepressor 2	−2.036	−1.649	−0.668	−0.753	−0.189	**−1.059**	0.012	1.933	+	yes
Q6IN85	2;2;2;0;0	3	**PPP4R3A**	Serine/threonine-protein phosphatase 4 regulatory subunit 3A	−1.542	−1.547	−0.091			**−1.060**	0.049	1.310	+	
Q5MIZ7	2;2;2;0;0	3	**PPP4R3B**	Serine/threonine-protein phosphatase 4 regulatory subunit 3B	−1.542	−1.547	−0.091			**−1.060**	0.049	1.310	+	
Q15035	1;1;1;0;0	3	**TRAM2**	Translocating chain-associated membrane protein 2	−1.218	−1.438	−0.531			**−1.062**	0.010	1.994	+	yes
Q6YHK3	1;1;1;0;0	3	**CD109**	CD109 antigen	−1.201	−1.013	−1.009			**−1.074**	0.001	3.214	+	yes
Q9NVP2	1;1;1;0;0	3	**ASF1B**	Histone chaperone ASF1B	−1.189	−1.355	−0.708			**−1.084**	0.004	2.419	+	yes
P83111	1;1;2;0;0	3	**LACTB**	Serine beta-lactamase-like protein LACTB, mitochondrial	−1.323	−1.158	−0.864			**−1.115**	0.001	2.824	+	yes
O75884	1;1;1;0;0	3	**RBBP9**	Serine hydrolase RBBP9	−1.263	−1.827	−0.263			**−1.118**	0.036	1.441	+	yes
Q9BXB4	1;1;2;1;1	5	**OSBPL11**	Oxysterol-binding protein-related protein 11	−2.613	−1.830	−0.608	−0.384	−0.315	**−1.150**	0.027	1.577	+	yes
Q9BVP2	6;6;5;1;1	5	**GNL3**	Guanine nucleotide-binding protein-like 3	−2.115	−2.149	−0.252	−0.448	−0.822	**−1.157**	0.017	1.773	+	yes
P15408	1;1;1;0;0	3	**FOSL2**	Fos-related antigen 2	−1.789	−1.325	−0.433			**−1.182**	0.021	1.681	+	yes
Q9NPL8	2;2;2;0;0	3	**TIMMDC1**	Complex I assembly factor TIMMDC1, mitochondrial	−1.706	−1.774	−0.210			−**1.230**	0.037	1.431	+	
P27448	1;1;1;0;0	3	**MARK3**	MAP/microtubule affinity-regulating kinase 3	−1.028	−1.553	−1.261			−**1.281**	0.001	2.925	+	
Q8NBN3	1;1;1;0;0	3	**TMEM87A**	Transmembrane protein 87A	−1.409	−0.523	−1.923			−**1.285**	0.017	1.760	+	
P24593	1;1;1;1;1	5	**IGFBP5**	Insulin-like growth factor-binding protein 5	−2.103	−0.357	−1.147	−2.098	−1.065	−**1.354**	0.004	2.438	+	yes
Q8WUJ3	2;2;5;3;3	5	**CEMIP**	Cell migration-inducing and hyaluronan-binding protein	−1.410	−0.411	−1.749	−1.766	−1.543	−**1.376**	0.001	3.060	+	
P78346	1;1;1;0;0	3	**RPP30**	Ribonuclease P protein subunit p30	−1.013	−2.101	−1.243			−**1.452**	0.006	2.205	+	yes
Q96S59	1;1;3;0;0	3	**RANBP9**	Ran-binding protein 9	−2.188	−2.144	−0.211			−**1.514**	0.040	1.398	+	
Q6VN20	1;1;3;0;0	3	**RANBP10**	Ran-binding protein 10	−2.188	−2.144	−0.211			−**1.514**	0.040	1.398	+	
Q9UI15	1;1;1;0;0	3	**TAGLN3**	Transgelin-3	−2.393	−0.583	−1.594			−**1.524**	0.021	1.677	+	yes
P31947	3;3;2;3;3	5	**SFN**	14-3-3 protein sigma	−1.689	−1.777	0.047	−2.983	−1.505	−**1.581**	0.009	2.040	+	yes
P35749	1;1;1;0;0	3	**MYH11**	Myosin-11	−2.266	−2.881	−0.241			−**1.796**	0.043	1.366	+	yes
Q13394	1;1;1;0;0	3	**MAB21L1**	Putative nucleotidyltransferase MAB21L1	−2.098	−1.064	−2.395			−**1.852**	0.005	2.300	+	
Q9Y586	1;1;1;0;0	3	**MAB21L2**	Protein mab-21-like 2	−2.098	−1.064	−2.395			−**1.852**	0.005	2.300	+	yes
Q5EBL8	1;1;1;0;0	3	**PDZD11**	PDZ domain-containing protein 11	−2.911	−2.010	−0.655			−**1.859**	0.022	1.654	+	
Q8NBM8	1;1;1;0;0	3	**PCYOX1L**	Prenylcysteine oxidase-like	−3.252	−2.370	−0.208			−**1.944**	0.049	1.313	+	yes
P27707	1;1;4;0;0	3	**DCK**	Deoxycytidine kinase	−3.758	−3.302	−0.182			−**2.414**	0.048	1.316	+	yes
O14556	2;2;0;1;1	4	**GAPDHS**	Glyceraldehyde-3-phosphate dehydrogenase, testis-specific	−2.243	−2.970		−2.681	−2.248	−**2.535**	0.000	4.571	+	
Q9UPN9	1;1;1;0;0	3	**TRIM33**	E3 ubiquitin-protein ligase TRIM33	−3.373	−4.084	−0.223			−**2.560**	0.048	1.322	+	yes
Q68CQ4	1;1;2;0;0	3	**UTP25**	U3 small nucleolar RNA-associated protein 25 homolog	−4.022	−2.963	−0.803			−**2.596**	0.024	1.620	+	
P16444	1;1;1;1;1	5	**DPEP1**	Dipeptidase 1	−5.281	−4.508	−2.807	−1.740	0.315	−**2.804**	0.017	1.777	+	yes
Q8IWY9	2;2;1;0;0	3	**CDAN1**	Codanin-1	−3.283	−2.659	−3.888			−**3.277**	0.000	3.356	+	yes

Differentially regulated protein groups in fibroblasts.

ISG = Interferon regulated gene.

*INTERFEROME database.

### Proteome analysis of endothelial cells exposed to Dense Bodies

**Fig. 2:**
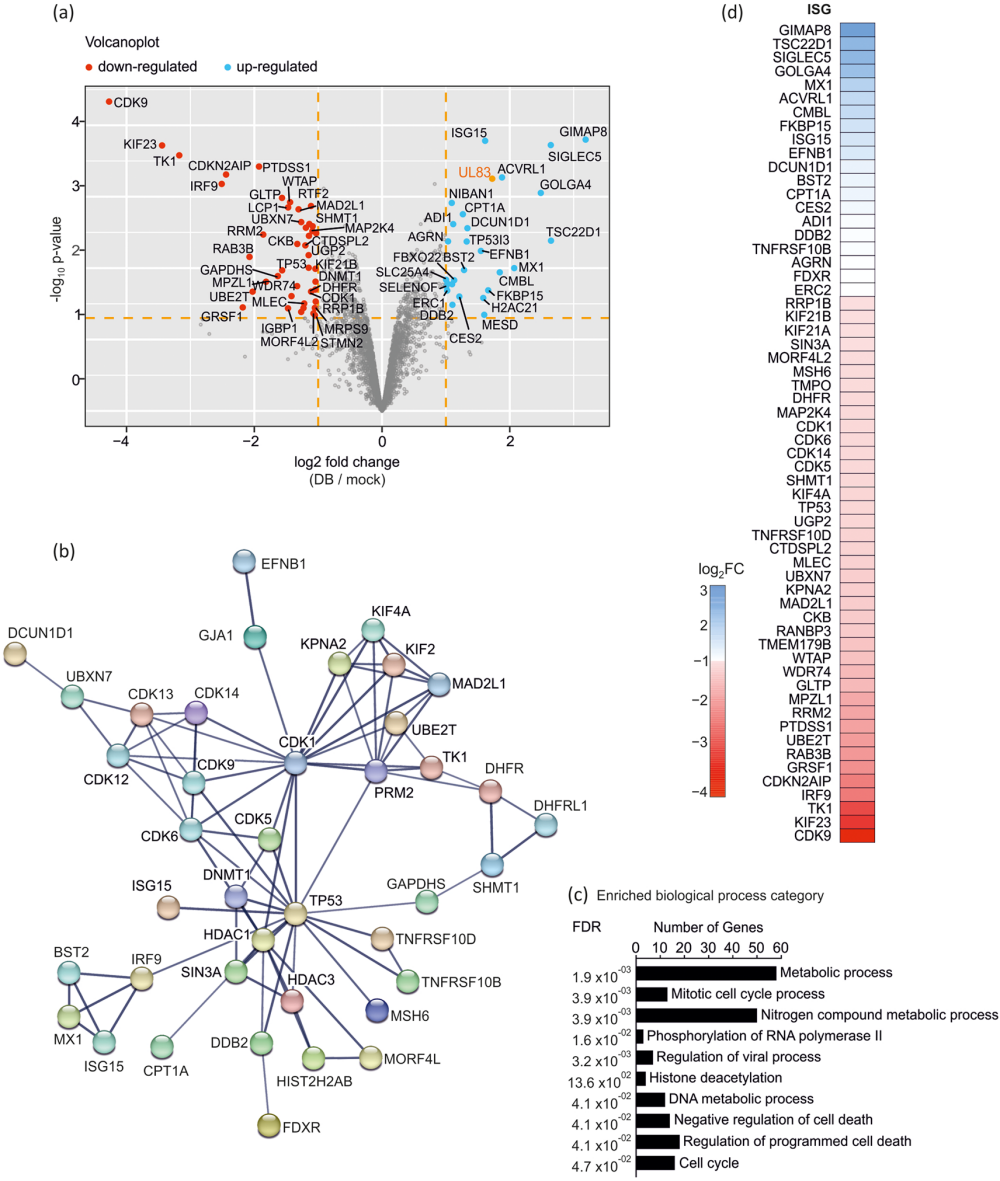
Quantitative proteomic analysis of differentially regulated proteins in DB-treated ECs.. Endothelial cells were incubated with 40 µg of UV-inactivated DB (strain TR-∆GFP) or left untreated. Cell lysates were prepared 24 h after application and subjected to proteome analysis. (**a**) The 2719 proteins identified by MS from three biological replicates are shown in a volcano plot according to their statistical p-value (y-axis) and their relative abundance ratio (log2 fold change) between DB- and mock-treated cells (x-axis). Red dots indicate differentially expressed proteins that were significantly downregulated after DB treatment (fold change > 1.0; p < 0.05). Blue dots indicate differentially expressed proteins that were significantly upregulated (fold change < 1.0; p < 0.05). The viral tegument protein pp65 (UL83) is highlighted in orange and was used as a control for DB internalisation into ECs. The volcano plot was generated using the R software. (**b**) STRING Protein-Protein Interaction network of the 83 proteins that were differentially expressed in ECs upon DB treatment. Proteins with no associations to other proteins in the network were removed. Network nodes represent all the proteins produced by a single, protein-coding gene locus. Lines depict protein interaction and the line thickness indicates the strength of the data support with a minimum confidence cut-off of 0.7 (high confidence). (**c**) Bar chart of the enriched biological processes associated with differentially expressed proteins. The top ten enriched biological processes are arranged according to increasing False Discovery Rates (FDR). The y-axis represents biological process categories, while the x-axis indicates the number of genes involved in each category. (**d**) 60 differentially regulated proteins were designated as IRGs. The log2FC is represented with a colour gradient. The 20 up-regulated ISGs are indicated in blue and the 40 down-regulated ISGs are indicated in red. STRING, Search Tool for the Retrieval of Interacting Genes.

Cellular proteins regulated upon exposure of Dense Bodies to endothelial cells were analysed from at least two biological replicates. On the basis of the filtering criteria previously used, 83 altered proteins (listed in Table 2) were found to be differentially regulated and are shown in the volcano plot in Fig. 2a. To identify the effects of DB-treatment on cellular pathways, the STRING database (https://string-db.org, accessed on 20.10.2022) was used. The PPI network in Fig. 2b shows the interactions between the 83 regulated proteins, using the high confidence interaction score of 0.7. Each of the differentially expressed proteins mapped to three or four major functional networks that were connected by the hub proteins CDK1 or TP53. One cluster is composed of the proteins MX1, ISG15, BST2 and IRF3 which are known to function in the type I interferon signalling pathway. The other two strongly connected networks comprise CDKs and KIF- proteins, both associated with the cell cycle. The top ten categories of biological processes that were enriched upon DB-application in endothelial cells are depicted in the bar chart in Fig. 2c and are arranged according to increasing False Discovery Rates (FDR). The 83 proteins were submitted to the Interferome database (v2.01, accessed November 2022)^25^. 60 proteins were identified as interferon stimulated genes, most of which were downregulated (Fig. 2d).

**Table 2:** Differentially expressed proteins identified in response to DB-treatment in endothelial cells.

UniProt ID	Ratio string	Replicate count	Gene name	Protein name	log2ratio			logFC	adjusted p-Value	minus_log10_p_value	significant	ISG
					repl 1	repl 2	repl 3					
Q8ND71	0;1;1	2	GIMAP8	GTPase IMAP family member 8		3.296	3.079	3.187	0.000	3.800	+	yes
Q15714	0;1;1	2	TSC22D1	TSC22 domain family protein 1		2.111	3.179	2.645	0.004	2.384	+	yes
O15389	0;2;2	2	SIGLEC5	Sialic acid-binding Ig-like lectin 5		2.655	2.628	2.642	0.000	3.723	+	yes
Q13439	0;1;1	2	GOLGA4	Golgin subfamily A member 4		2.240	2.731	2.486	0.001	3.053	+	yes
P20591	18;27;27	3	MX1	Interferon-induced GTP-binding protein Mx1	3.312	1.393	1.504	2.069	0.010	2.000	+	yes
P37023	0;1;1	2	ACVRL1	Serine/threonine-protein kinase receptor R3		1.846	1.911	1.879	0.001	3.270	+	yes
Q96DG6	5;5;5	3	CMBL	Carboxymethylenebutenolidase homolog	0.710	2.579	2.239	1.843	0.011	1.940	+	yes
P18139	4;10;10	3	HCMV UL83	65 kDa phosphoprotein	2.064	1.778	1.332	1.725	0.001	3.252	+	
Q5T1M5	0;1;1	2	FKBP15	FK506-binding protein 15		2.264	1.059	1.662	0.020	1.689	+	yes
P05161	7;10;10	3	ISG15	Ubiquitin-like protein ISG15	1.825	1.413	1.605	1.614	0.000	3.783	+	yes
Q14696	2;2;2	3	MESD	LRP chaperone MESD	0.073	2.138	2.589	1.600	0.045	1.346	+	
Q8IUE6	2;1;1	3	H2AC21	Histone H2A type 2-B	0.326	2.039	2.390	1.585	0.026	1.582	+	
P98172	0;1;1	2	EFNB1	Ephrin-B1		1.231	1.864	1.548	0.006	2.241	+	yes
Q96GG9	0;1;1	2	DCUN1D1	DCN1-like protein 1		1.489	1.184	1.336	0.003	2.559	+	yes
Q53FA7	7;10;10	3	TP53I3	Quinone oxidoreductase PIG3	0.732	1.642	1.602	1.325	0.004	2.372	+	
Q10589	2;1;1	3	BST2	Bone marrow stromal antigen 2	0.777	2.034	1.041	1.284	0.011	1.974	+	yes
P50416	0;2;2	2	CPT1A	Carnitine O-palmitoyltransferase 1, liver isoform		1.308	1.224	1.266	0.002	2.755	+	yes
O00748	0;2;2	2	CES2	Cocaine esterase		0.753	1.668	1.210	0.025	1.602	+	yes
Q8NEZ5	2;1;1	3	FBXO22	F-box only protein 22	0.393	1.551	1.444	1.129	0.015	1.831	+	
Q9BV57	0;2;2	2	ADI1	Acireductone dioxygenase		1.097	1.131	1.114	0.002	2.616	+	yes
Q92466	3;1;1	3	DDB2	DNA damage-binding protein 2	0.181	1.726	1.400	1.102	0.033	1.486	+	yes
O14763	1;3;3	3	TNFRSF10B	Tumor necrosis factor receptor superfamily member 10B	1.844	0.750	0.698	1.097	0.017	1.775	+	yes
Q9BZQ8	2;1;1	3	NIBAN1	Protein Niban 1	0.916	1.387	0.972	1.092	0.001	2.913	+	
O00468	0;1;1	2	AGRN	Agrin		1.137	0.929	1.033	0.004	2.375	+	yes
P22570	19;13;13	3	FDXR	NADPH:adrenodoxin oxidoreductase, mitochondrial	0.336	1.378	1.357	1.023	0.016	1.783	+	yes
Q8IUD2	0;4;4	2	ERC1	ELKS/Rab6-interacting/CAST family member 1		1.357	0.684	1.020	0.021	1.686	+	yes
O15083	0;4;4	2	ERC2	ERC protein 2		1.357	0.684	1.020	0.021	1.686	+	yes
O60613	0;1;1	2	SELENOF	Selenoprotein F		0.701	1.314	1.007	0.018	1.749	+	
P12235	0;2;2	2	SLC25A4	ADP/ATP translocase 1		0.729	1.276	1.003	0.015	1.837	+	
P17302	0;2;2	2	GJA1	Gap junction alpha-1 protein		−0.985	−1.079	−1.032	0.003	2.490	+	
Q14684	2;1;1	3	RRP1B	Ribosomal RNA processing protein 1 homolog B	−0.220	−1.249	−1.647	−1.038	0.029	1.532	+	yes
P26358	2;2;2	3	DNMT1	DNA (cytosine-5)-methyltransferase 1	−0.424	−1.091	−1.610	−1.042	0.015	1.810	+	
O75037	0;1;1	2	KIF21B	Kinesin-like protein KIF21B		−0.807	−1.281	−1.044	0.010	1.991	+	yes
Q7Z4S6	0;1;1	2	KIF21A	Kinesin-like protein KIF21A		−0.807	−1.281	−1.044	0.010	1.991	+	yes
Q96ST3	1;1;1	3	SIN3A	Paired amphipathic helix protein Sin3a	−0.320	−1.575	−1.237	−1.044	0.020	1.700	+	yes
Q93045	1;3;3	3	STMN2	Stathmin-2	−0.058	−1.395	−1.698	−1.050	0.046	1.340	+	
P82933	0;3;2	2	MRPS9	28 S ribosomal protein S9, mitochondrial		−0.594	−1.510	−1.052	0.036	1.443	+	
Q15014	0;1;1	2	MORF4L2	Mortality factor 4-like protein 2		−1.583	−0.564	−1.074	0.043	1.365	+	yes
P52701	0;4;4	2	MSH6	DNA mismatch repair protein Msh6		−1.071	−1.102	−1.087	0.003	2.586	+	yes
P42166	0;2;2	2	TMPO	Lamina-associated polypeptide 2, isoform alpha		−1.161	−1.019	−1.090	0.003	2.517	+	yes
P00374	2;4;4	3	DHFR	Dihydrofolate reductase	−0.336	−1.674	−1.294	−1.101	0.020	1.698	+	yes
Q86XF0	2;4;4	3	DHFR2	Dihydrofolate reductase 2, mitochondrial	−0.336	−1.674	−1.294	−1.101	0.020	1.698	+	
P45985	0;1;1	2	MAP2K4	Dual specificity mitogen-activated protein kinase kinase 4		−1.014	−1.189	−1.102	0.003	2.495	+	
P06493	10;11;11	3	CDK1	Cyclin-dependent kinase 1	−0.297	−1.433	−1.603	−1.111	0.021	1.671	+	yes
Q00534	10;11;11	3	CDK6	Cyclin-dependent kinase 6	−0.297	−1.433	−1.603	−1.111	0.021	1.671	+	yes
Q96Q40	10;11;11	3	CDK15	Cyclin-dependent kinase 15	−0.297	−1.433	−1.603	−1.111	0.021	1.671	+	
O94921	10;11;11	3	CDK14	Cyclin-dependent kinase 14	−0.297	−1.433	−1.603	−1.111	0.021	1.671	+	yes
Q00535	10;11;11	3	CDK5	Cyclin-dependent kinase 5	−0.297	−1.433	−1.603	−1.111	0.021	1.671	+	yes
Q9NYV4	10;11;11	3	CDK12	Cyclin-dependent kinase 12	−0.297	−1.433	−1.603	−1.111	0.021	1.671	+	
Q14004	10;11;11	3	CDK13	Cyclin-dependent kinase 13	−0.297	−1.433	−1.603	−1.111	0.021	1.671	+	
Q9BY42	2;2;2	3	RTF2	Replication termination factor 2	−0.878	−1.042	−1.416	−1.112	0.001	2.870	+	
P34896	0;2;2	2	SHMT1	Serine hydroxymethyltransferase, cytosolic		−1.182	−1.094	−1.138	0.002	2.618	+	yes
O95239	1;2;2	3	KIF4A	Chromosome-associated kinesin KIF4A	−0.677	−1.420	−1.336	−1.144	0.004	2.452	+	yes
P04637	6;3;3	3	TP53	Cellular tumor antigen p53	−0.491	−1.416	−1.538	−1.149	0.010	2.005	+	yes
Q16851	0;1;1	2	UGP2	UTP–glucose-1-phosphate uridylyltransferase		−0.936	−1.363	−1.149	0.007	2.181	+	yes
Q9UBN6	0;2;2	2	TNFRSF10D	Tumor necrosis factor receptor superfamily member 10D		−1.093	−1.292	−1.192	0.003	2.566	+	yes
Q05D32	0;1;1	2	CTDSPL2	CTD small phosphatase-like protein 2		−1.014	−1.387	−1.201	0.005	2.318	+	yes
Q14165	3;1;1	3	MLEC	Malectin	−0.263	−2.030	−1.364	−1.219	0.031	1.504	+	yes
Q13547	1;1;1	3	HDAC1	Histone deacetylase 1	−0.559	−2.312	−0.818	−1.230	0.036	1.441	+	
O15379	1;1;1	3	HDAC3	Histone deacetylase 3	−0.559	−2.312	−0.818	−1.230	0.036	1.441	+	
O94888	0;1;1	2	UBXN7	UBX domain-containing protein 7		−1.168	−1.364	−1.266	0.002	2.645	+	yes
P52292	2;6;6	3	KPNA2	Importin subunit alpha-1	−0.089	−1.813	−1.896	−1.266	0.041	1.386	+	yes
Q13257	0;2;2	2	MAD2L1	Mitotic spindle assembly checkpoint protein MAD2A		−1.307	−1.306	−1.307	0.002	2.823	+	yes
P12277	1;2;2	3	CKB	Creatine kinase B-type	−0.720	−1.681	−1.580	−1.327	0.005	2.338	+	yes
Q9H6Z4	3;3;3	3	RANBP3	Ran-binding protein 3	−0.393	−1.845	−1.750	−1.329	0.018	1.748	+	yes
Q7Z7N9	0;1;1	2	TMEM179B	Transmembrane protein 179B		−1.964	−0.872	−1.418	0.025	1.608	+	yes
Q15007	0;1;1	2	WTAP	Pre-mRNA-splicing regulator WTAP		−1.401	−1.478	−1.439	0.001	2.923	+	yes
P13796	0;1;1	2	LCP1	Plastin-2		−1.562	−1.378	−1.470	0.001	2.850	+	
P78318	1;1;1	3	IGBP1	Immunoglobulin-binding protein 1	−0.150	−2.129	−2.138	−1.472	0.037	1.437	+	
Q6RFH5	0;1;1	2	WDR74	WD repeat-containing protein 74		−1.136	−1.992	−1.564	0.011	1.969	+	yes
Q9NZD2	0;2;2	2	GLTP	Glycolipid transfer protein		−1.498	−1.634	−1.566	0.001	2.984	+	yes
O14556	0;2;2	2	GAPDHS	Glyceraldehyde-3-phosphate dehydrogenase, testis-specific		−2.119	−1.144	−1.631	0.013	1.889	+	
O95297	0;2;2	2	MPZL1	Myelin protein zero-like protein 1		−2.409	−1.222	−1.816	0.016	1.809	+	yes
P31350	0;3;3	2	RRM2	Ribonucleoside-diphosphate reductase subunit M2		−2.177	−1.543	−1.860	0.003	2.470	+	yes
P48651	1;1;1	3	PTDSS1	Phosphatidylserine synthase 1	−1.734	−2.356	−1.689	−1.926	0.000	3.422	+	yes
Q9NPD8	0;1;1	2	UBE2T	Ubiquitin-conjugating enzyme E2 T		−2.787	−1.265	−2.026	0.021	1.670	+	yes
P20337	0;2;2	2	RAB3B	Ras-related protein Rab-3B		−2.574	−1.577	−2.075	0.007	2.157	+	yes
Q12849	1;1;1	3	GRSF1	G-rich sequence factor 1	−0.268	−2.787	−3.488	−2.181	0.036	1.450	+	yes
Q9NXV6	0;1;1	2	CDKN2AIP	CDKN2A-interacting protein		−2.284	−2.599	−2.442	0.000	3.311	+	yes
Q00978	0;1;1	2	IRF9	Interferon regulatory factor 9		−2.720	−2.300	−2.510	0.001	3.179	+	yes
P04183	0;4;4	2	TK1	Thymidine kinase, cytosolic		−3.354	−2.996	−3.175	0.000	3.580	+	yes
Q02241	0;1;1	2	KIF23	Kinesin-like protein KIF23		−3.613	−3.275	−3.444	0.000	3.718	+	yes
P50750	0;2;2	2	CDK9	Cyclin-dependent kinase 9		−4.304	−4.243	−4.273	0.000	4.332	+	yes

Differentially regulated protein groups in endothelial cells.

ISG = Interferon regulated gene.

The validation of the MS-analyses provided here was published elsewhere^11^. In that study, we evaluated the expression of the selected proteins MX1, IFIT3 and ISG15 in fibroblasts and endothelial cells using Western blot analyses. The Western blot results were consistent with the results obtained from the MS data. A robust increase in the expression levels of all three proteins upon the DB-treatment could be confirmed. Although the fold changes were not identical in the immunoblot analyses, compared to the MS data at 24 h.p.a., the tendencies were similar. Taken together, these experimental results show that our proteomics data are reliable.

## Data Records

### Data record 1

All mass spectrometry raw data, fasta files and direct output text files from MaxQuant analysis are available on the Proteomics Identifications (PRIDE) partner repository^20^ (http://www.ebi.ac.uk/pride/archive/projects/PXD039032)^26^.

## Technical Validation

### Virus consistency for DB production

To enable consistency between the different DB preparations, HFF of the same passage were infected with the same batch of the HCMV TR-∆GFP supernatant-stock.

### Dense bodies internalization control

After each preparation, purified Dense Bodies were tested for their ability to enter cells. For this, a nuclear staining of the viral major tegument protein pp65 was taken as evidence for entry into cells^26,27^. 2 × 10^5^ HFF or ECs were grown on coverslips and incubated with 5 µg (HFF) or 10 µg (ECs) DB of TR-∆GFP and analysed by immunofluorescence microscopy one day post application. Cells were fixed with 90% acetone. The nuclear localization of DB-derived pp65 was detected using a pp65-specific monoclonal antibody and an anti-mouse Alexa Fluor® 546-conjugate secondary antibody. Cell nuclei were counterstained with DAPI (Sigma-Aldrich). Visualization was done by fluorescence microscopy with an Axiophot microscope equipped with a SPOT Flex camera FX1520 (Zeiss, Jena, Germany).

### Reproducibility within the replicates

**Fig. 3:**
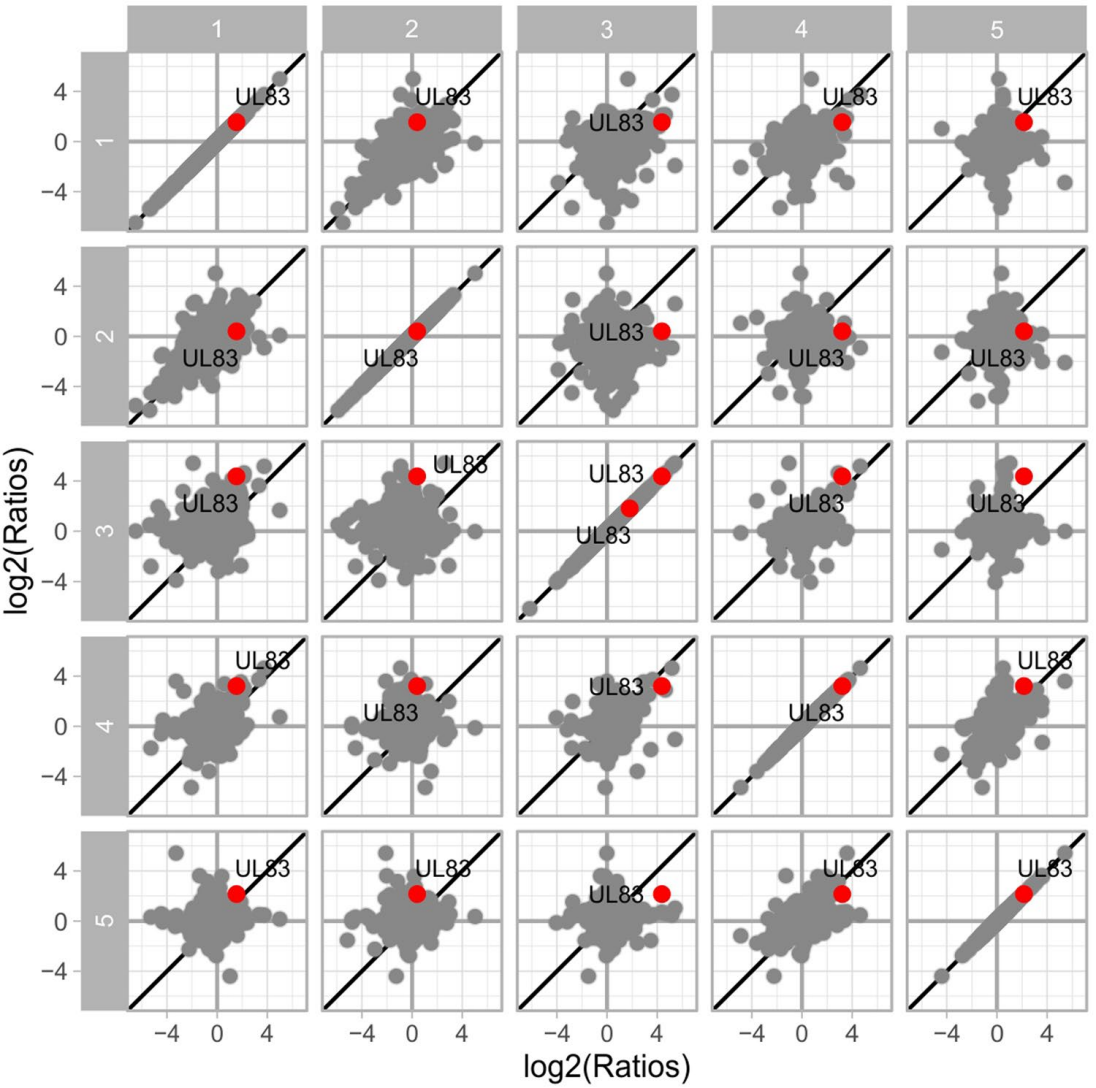
Reproducibility of detected protein log2 ratios of DB-treated vs. control in five replicates of fibroblasts. The diagonal indicates perfect alignment. The viral protein UL83 is upregulated as expected. Note that two different isoforms of UL83 protein was detected in replicate 3.

**Fig. 4:**
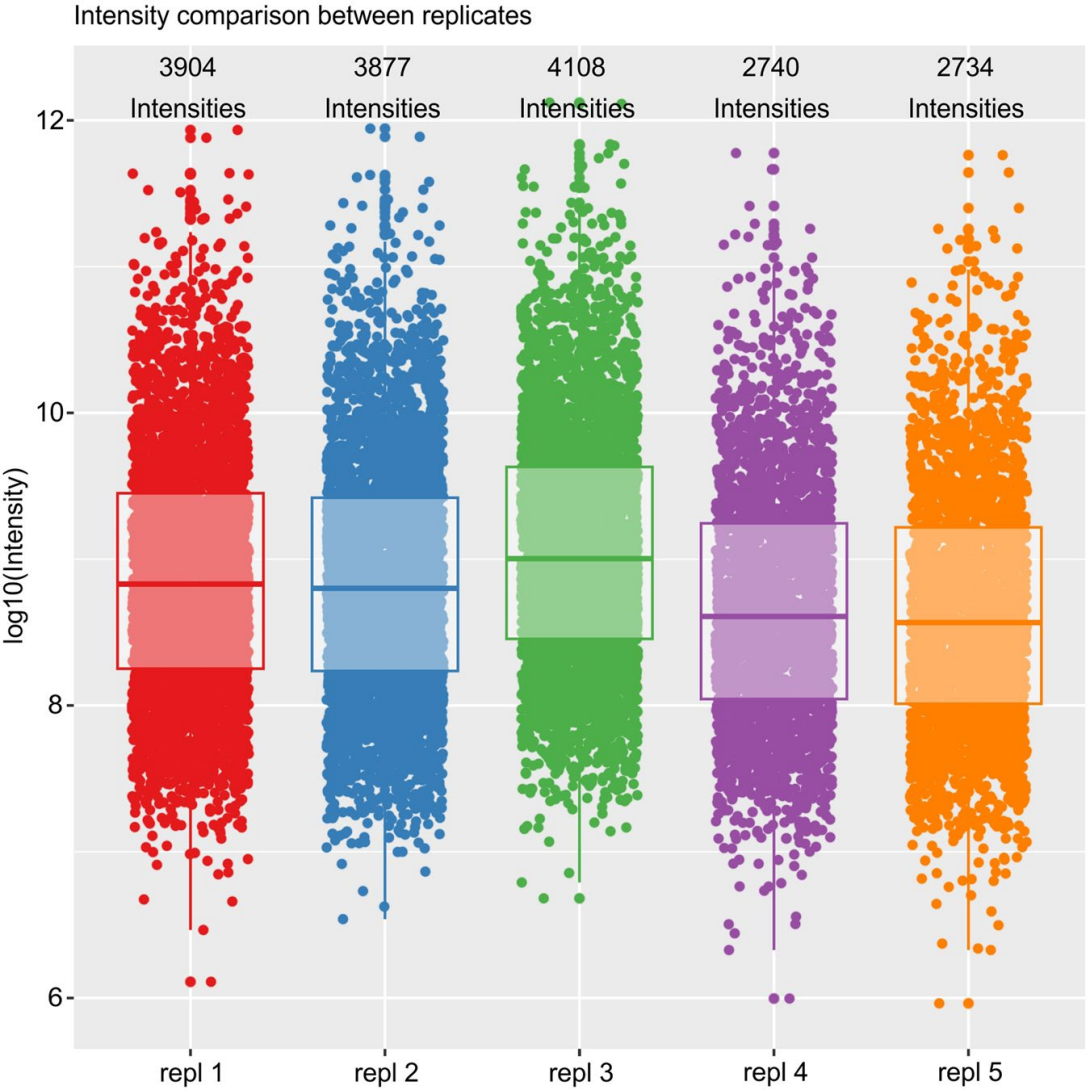
Detected protein intensities of DB-treated vs. control in the five replicates of fibroblasts. Each box represents median and interquartile range.

**Fig. 5:**
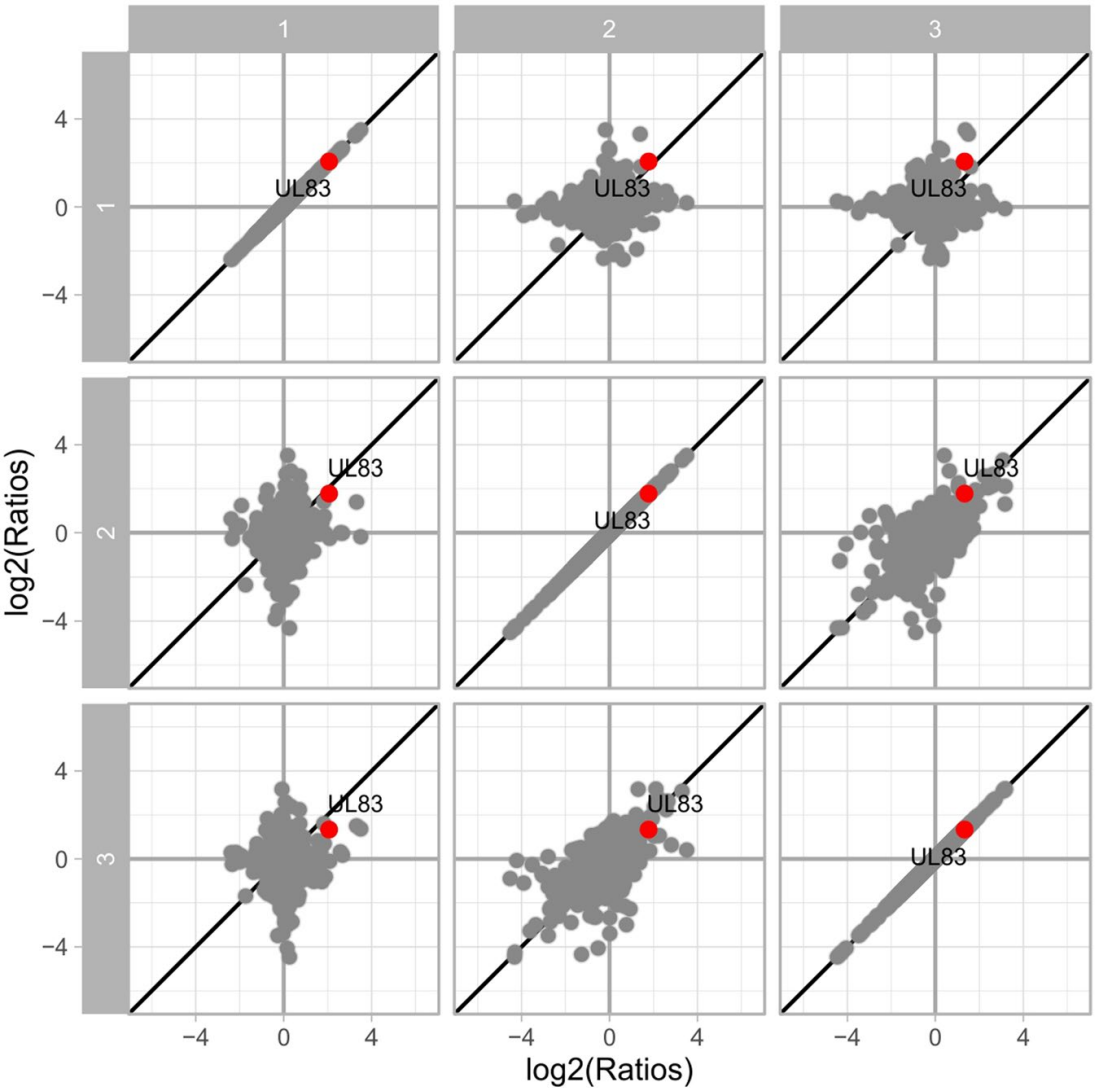
Reproducibility of detected protein log2 ratios of DB-treated vs. control in three replicates of endothelial cells. The diagonal indicates perfect alignment. The viral protein UL83 is confirmed upregulated as expected.

To account for potential technical and biological variation, the study was performed using both technical replicates and multiple biological replicates (5 for fibroblasts; 2 for endothelial cells). The regulation of protein abundance induced upon HCMV DB treatment was generally consistent across biological replicates in both cell types, especially with regard to the induction of repression of individual proteins (Figs. 3, 4 and 5). As described in the methods section, we performed multiple steps of stringency filtering of the data. To identify proteins that were consistently regulated across biological replicates, we used the linear modelling to quantify the significance of the regulation. Due to batch effect, we observed fewer quantification events and lower intensities for replicates 4 and 5, as compared to the first three replicates. In spite of that, the isotope-labelling approach using dimethyl-labelling rendered the comparison between conditions robust within each replicate (Fig. 3). Nevertheless, we further increased the stringency filtering requirement from detection in two out of five replicates to at least three in the fibroblast dataset.

### Supplementary information

Analysis_paper R (supplementary file)

## Data Availability

Data analysis procedures have been described in detail in the Methods section. Code for the statistical analysis performed in R is available as a supplement file.

## References

[1] Boppana, S. B. & Britt, W. J. in *Cytomegaloviruses From Molecular Pathogenesis to Intervention* Vol. 2 (ed Reddehase, M. J.) Ch. II.1, 1–25 (Caister Academic Press, 2013).

[2] Sarov I, Abady I (1975). The morphogenesis of human cytomegalovirus. Isolation and polypeptide characterization of cytomegalovirions and dense bodies. Virology.

[3] Craighead JE, Kanich RE, Almeida JD (1972). Nonviral microbodies with viral antigenicity produced in cytomegalovirus-infected cells. J. Virol.

[4] Varnum SM (2004). Identification of proteins in human cytomegalovirus (HCMV) particles: the HCMV proteome. J. Virol.

[5] Büscher N, Paulus C, Nevels M, Tenzer S, Plachter B (2015). The proteome of human cytomegalovirus virions and dense bodies is conserved across different strains. Med. Microbiol. Immunol.

[6] Pepperl S, Münster J, Mach M, Harris JR, Plachter B (2000). Dense bodies of human cytomegalovirus induce both humoral and cellular immune responses in the absence of viral gene expression. J. Virol.

[7] Lehmann, C. *et al*. Dense bodies of a gH/gL/UL128-131 pentamer repaired Towne strain of human cytomegalovirus induce an enhanced neutralizing antibody response. *J Virol* **93**, 10.1128/JVI.00931-19 (2019). 10.1128/JVI.00931-19 PMC6694834 31189713

[8] Gergely KM (2021). Therapeutic Vaccination of Hematopoietic Cell Transplantation Recipients Improves Protective CD8 T-Cell Immunotherapy of Cytomegalovirus Infection. Front Immunol.

[9] Becke S (2010). Optimized recombinant dense bodies of human cytomegalovirus efficiently prime virus specific lymphocytes and neutralizing antibodies without the addition of adjuvant. Vaccine.

[10] Cayatte C (2013). Cytomegalovirus vaccine strain towne-derived dense bodies induce broad cellular immune responses and neutralizing antibodies that prevent infection of fibroblasts and epithelial cells. J Virol.

[11] Penner, I. *et al*. Subviral Dense Bodies of Human Cytomegalovirus Induce an Antiviral Type I Interferon Response. *Cells* **11**, 10.3390/cells11244028 (2022). 10.3390/cells11244028 PMC9777239 36552792

[12] Schleiss MR (2008). Cytomegalovirus vaccine development. Curr. Top. Microbiol. Immunol.

[13] Plotkin, S. A. & Plachter, B. in *Cytomegaloviruses: From Molecular Pathogenesis to Intervention* (ed Reddehase, M. J.) Ch. 2.20, 424–449 (Caister Academic Press, 2013).

[14] Frankenberg N, Lischka P, Pepperl-Klindworth S, Stamminger T, Plachter B (2012). Nucleocytoplasmic shuttling and CRM1-dependent MHC class I peptide presentation of human cytomegalovirus pp65. Med. Microbiol. Immunol.

[15] Besold K (2007). Processing and MHC class I presentation of human cytomegalovirus pp65-derived peptides persist despite gpUS2-11-mediated immune evasion. J. Gen. Virol.

[16] Besold K, Wills M, Plachter B (2009). Immune evasion proteins gpUS2 and gpUS11 of human cytomegalovirus incompletely protect infected cells from CD8 T cell recognition. Virology.

[17] May T (2010). Synthetic gene regulation circuits for control of cell expansion. Tissue Eng Part A.

[18] Lieber, D. *et al*. A permanently growing human endothelial cell line supports productive infection with human cytomegalovirus under conditional cell growth arrest. *Biotechniques* **59**, 127–136, 10.2144/000114326 (2015). 10.2144/000114326 26345505

[19] Goldner, T. *et al*. The novel anticytomegalovirus compound AIC246 (Letermovir) inhibits human cytomegalovirus replication through a specific antiviral mechanism that involves the viral terminase. *J. Virol* **85**, 10884–10893, 10.1128/JVI.05265-11 (2011). 10.1128/JVI.05265-11 PMC3187482 21752907

[20] Perez-Riverol Y (2022). The PRIDE database resources in 2022: a hub for mass spectrometry-based proteomics evidences. Nucleic Acids Res.

[21] Rappsilber J, Ishihama Y, Mann M (2003). Stop and go extraction tips for matrix-assisted laser desorption/ionization, nanoelectrospray, and LC/MS sample pretreatment in proteomics. Anal Chem.

[22] Cox J, Mann M (2008). MaxQuant enables high peptide identification rates, individualized p.p.b.-range mass accuracies and proteome-wide protein quantification. Nat Biotechnol.

[23] Cox J (2011). Andromeda: a peptide search engine integrated into the MaxQuant environment. J Proteome Res.

[24] Phipson B, Lee S, Majewski IJ, Alexander WS, Smyth GK (2016). Robust Hyperparameter Estimation Protects against Hypervariable Genes and Improves Power to Detect Differential Expression. Ann Appl Stat.

[25] Rusinova I (2013). Interferome v2.0: an updated database of annotated interferon-regulated genes. Nucleic Acids Res.

[26] Penner I (2022). Proteome changes of fibroblasts and endothelial cells upon incubation with human cytomegalovirus Dense Bodies..

[27] Gogesch, P. *et al*. Production Strategies for Pentamer-Positive Subviral Dense Bodies as a Safe Human Cytomegalovirus Vaccine. *Vaccines (Basel)* **7**, 10.3390/vaccines7030104 (2019). 10.3390/vaccines7030104 PMC6789746 31480520

